# Effect of Dietary Fat Supplementation during Late Pregnancy and First Six Months of Lactation on Maternal and Infant Vitamin A Status in Rural Bangladesh

**DOI:** 10.3329/jhpn.v28i4.6039

**Published:** 2010-08

**Authors:** Dewan S. Alam, Joop M.A. van Raaij, Joseph G.A.J. Hautvast, M. Yunus, M.A. Wahed, G.J. Fuchs

**Affiliations:** ^1^ ICDDR,B, Mohakhali, Dhaka 1212, Bangladesh; ^2^ Division of Human Nutrition and Epidemiology, Wageningen University, Wageningen, The Netherlands; ^3^ Health and Nutrition Wing, Hodavasi Chawdhury & Company, Dhaka, Bangladesh; ^4^ University of Arkansas for Medical Sciences, Arkansas Children's Hospital, 800 Marshall St Ste 653, Little Rock, AR, USA

**Keywords:** Community-based studies, Fat supplementation, Infant, Postpartum;Pregnancy, Vitamin A, Vitamin A deficiency, Bangladesh

## Abstract

Dietary fat intake is extremely low in most communities with vitamin A deficiency. However, its role in vitamin A status of pregnant and lactating women is poorly understood. The aim of the study was to examine the effect of supplementing women with fat from mid-/late pregnancy until six months postpartum on their vitamin A status and that of their infants. Women recruited at 5-7 months of gestation were supplemented daily with 20 mL of soybean-oil (n=248) until six months postpartum or received no supplement (n=251). Dietary fat intake was assessed by 24-hour dietary recall at enrollment and at 1, 3 and 6 months postpartum. Concentrations of maternal plasma retinol, β-carotene, and lutein were measured at enrollment and at 1, 3 and 6 months postpartum, and those of infants at six months postpartum. Concentration of breastmilk retinol was measured at 1, 3 and 6 months postpartum. The change in concentration of plasma retinol at three months postpartum compared to pregnancy was significantly higher in the supplemented compared to the control women (+0.04 vs -0.07 μmol/L respectively; p<0.05)*.* Concentrations of plasma β-carotene and lutein declined in both the groups during the postpartum period but the decline was significantly less in the supplemented than in the control women at one month (β-carotene -0.07 vs -0.13 μmol/L, p<0.05); lutein -0.26 vs -0.49 μmol/L, p<0.05) and three months (β-carotene -0.04 vs -0.08 μmol/L, p<0.05; lutein -0.31 vs -0.47 μmol/L, p<0.05). Concentration of breastmilk retinol was also significantly greater in the supplemented group at three months postpartum than in the controls (0.68±0.35 vs 0.55±0.34 μmol/L respectively, p<0.03). Concentrations of infants’ plasma retinol, β-carotene, and lutein, measured at six months of age, did not differ between the groups. Fat supplementation during pregnancy and lactation in women with a very low intake of dietary fat has beneficial effects on maternal postpartum vitamin A status.

## INTRODUCTION

Vitamin A deficiency among pregnant and lactating women is widely prevalent in many developing countries (1–3) and poses serious threat to public health because of implications for maternal health and survival (4). Maternal vitamin A status has potential implications for the vitamin A status of their foetus and breastfed infants (5).

Conventional approaches to combat vitamin A deficiency include periodic supplementation with vitamin A, fortification of foods, and a food-based approach with increased consumption of dark green-leafy vegetables. Among the intervention strategies, the dietary approach with an emphasis on increased consumption of provitamin A carotenoid-containing foods has been advocated as a preferable and sustainable strategy to eliminate vitamin A deficiency (6,7). However, the effectiveness of this approach has been questioned as recent evidence suggested the poorer bioavailability of provitamin A plant carotenoids (8–12). In the late 1990s, the Food and Nutrition Board and the Institute of Medicine revised the conversion factor of β-carotene to retinol equivalent from 6:1 to 12:1 (13). More recent data suggested 21:1 as the conversion factor for β-carotene in mixed meal and that for vegetables as low as 26:1 (14). A study on healthy volunteers supplemented with purified stable isotope-labelled β-carotene in oil reported the conversion factor of 9.1 to 1 by weight (15). Data from Bangladesh showed lower conversion of β-carotene obtained from sweet potato (13:1) than synthetic β-carotene (6:1), suggesting the poorer bioavailability of β-carotene of vegetable origin (16). The bioavailability of provitamin A carotenoids is further constrained by other dietary factors, most notably dietary fat intake which is essential for optimal absorption and is characteristically low in most populations with vitamin A deficiency (17–19).

Studies to date investigating the relationship between dietary fat and vitamin A status have been limited to investigations of efficacy in highly-controlled settings with a relatively-small number of subjects. Studies in children have shown that fat supplementation enhances the absorption of β-carotene and improves the vitamin A status (18). A study in Indonesia demonstrated a significant improvement in the vitamin A status in vitamin A- deficient children supplemented with carotenoid-rich foods and high fat (18 g/day) compared to those supplemented with similar foods but with low fat (only 3 g/day). In adults, a significantly-greater concentration of plasma β-carotene was observed in those who received a high-fat supplementation (>60 g/day) than those consuming a low-fat (<8 g/day) diet. Results of a study in healthy adults in the Netherlands showed an improvement in concentration of plasma β-carotene in fat-supplemented groups compared to controls but no difference was observed between low- and high-fat (3 vs 36 g) supplementation (20). However, a more recent study reported higher absorption of carotenoids with increased fat content in fresh salad dressing (21). Although the amount of fat in diet for optimal absorption of carotenoids is still debated, supplementation appears to improve the absorption of carotenoids and/or vitamin A status in small-scale studies. However, population-based data are lacking and, to our knowledge, are non-existent on pregnant and lactating women. In this paper, we present the findings of a controlled intervention trial in which women in mid-/late pregnancy and throughout the first six months of lactation received either a daily supplement of 18 g of fat or no supplement.

## MATERIALS AND METHODS

### Population and study design

This community-based controlled trial was conducted from November 1995 to October 1997 in 16 villages in Matlab upazila (subdistrict) of Chandpur district, Bangladesh. The area, located approximately 55 km southeast of the capital city of Bangladesh—Dhaka, considered to be typical of rural and riverine delta areas (22). The usual diet in rural Bangladesh is known to be monotonous and low in fat (17). Rice is the main staple food usually eaten with green-leafy vegetables and a small amount of fish. Consumption of meat and other animal products is very seldom.

Sixteen socioeconomically-similar villages were grouped into two sets of eight each, separated by a distance of about 2 km. One set of villages was randomly selected for dietary intervention. It was felt that randomization of half of each selected area will have the same effect as would individual randomization in that both intervention and control groups will come from the same geographical area and community as each other. Therefore, any area-level effects are accounted for by the research design. The study participants were healthy pregnant women in their early or mid-pregnancy identified through menstrual history. A survey was conducted in the study villages to identify all currently-eligible women and also to list all married women of childbearing age, who could potentially become pregnant. A field team consisting of a health assistant, a community health worker (CHW), a dietary interviewer (a CHW with graduate-level education specially trained for dietary interviews), and a porter visited each eligible subject at home and explained the study and meaning of their voluntary participation. A detailed interview on socioeconomic, demographic and household characteristics was conducted. Trained and experienced field workers measured body-weight, height, mid-upper arm circumference (MUAC) following standard procedures (23). Data on food intake and blood specimens were collected at baseline and at 1, 3 and 6 months postpartum when specimens of breastmilk were also collected. A single blood specimen from each infant was collected between 6 and 7 months of age.

**Fig. 1.**
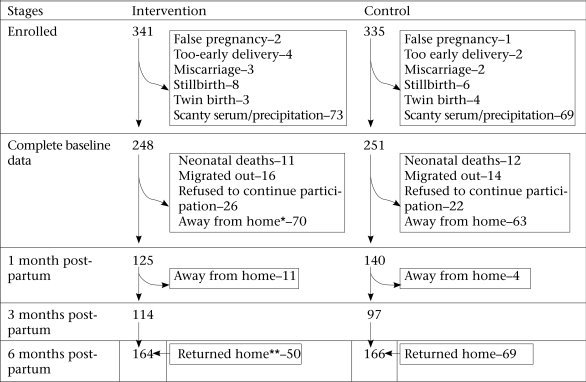
Study flow-chart

During the study period, 341 and 335 women were recruited from the intervention and control villages respectively (Fig. 1). From the total sample, 137 subjects were lost to follow-up, including 3 false pregnancies, 5 miscarriages, 6 too-early delivery (delivered less than one month after enrollment), 14 stillbirths, 7 twin births, 23 neonatal deaths, and 30 outmigrations; 48 refused to continue participation. Data of women who delivered singleton babies available during the postpartum period were included in the analysis. In total, 326 women in the intervention and 315 women in the control villages respectively delivered live singleton infants. However, 248 women from the intervention group and 251 from the control group had complete baseline data. Missing data at baseline were mainly biochemical ones which could not be obtained due to scant amount or precipitation of serum samples.

*The participants who were away from home during the scheduled visit. Over 85% of such women received oil supply (either received the ration in advance or delivered if they were visiting relatives in the neighbouring villages); **The participants who returned home and participated again

### Intervention

The intervention consisted of unfortified soybean-oil supplied to the women in the intervention villages. The women in the control villages were not given any other dietary supplement or advice and maintained their usual diet. Each woman in the intervention villages was supplied with a weekly ration of 140 mL of soybean-oil and was asked to consume 10 mL of oil twice a day (total 20 mL or ∼18 g fat) with their main meals (usually afternoon and night meals) as supplement. The community health worker (CHW) delivered the oil in a plastic bottle and a clearly-marked (at 10 mL) plastic dispenser to ensure the dose to each study woman in the home. Women were given several options to consume the supplement that included mixing of the oil with their foods, drinking during the meal, or frying their portion of the meal with the oil. However, pre-testing revealed that drinking during the meal was the most preferred choice of supplement intake, followed by mixing the oil directly with their meal portion. The study was, therefore, designed to supplement the diet in this way rather than instructing women to add oil during preparation of household food to ensure a measured ‘dose’ of oil for more accurate quantification of supplement intake. The total period of oil supplementation in an individual woman ranged from 8 to 10 months depending on the gestational ages of women at enrollment. The participants in the control group were not given any extra food to balance energy between the groups.

Motivational efforts were continued throughout the study period to maximize high compliance to the intervention and to avoid any replacement of the usual diet by the supplement. Each subject was trained to maintain the daily record of oil intake using an easily-understandable record-keeping sheet supplied each time the oil was delivered. The CHWs collected the records of oil intake during their next weekly home-visits, and they supplied the participant with another bottle of oil for the ensuing week. They also interviewed the subject and obtained information about her attitudes towards and compliance to the oil during the previous one week.

### Food and nutrient intake

Fat intake from all sources was estimated by 24-hour dietary recall administered four times during the study period following standard guidelines (23). A survey was conducted to obtain representative recipes for multi-ingredient foods consumed in the community. All locally-used household measures (plates, cup, spoons, etc.) were standardized for respective food items during the initial phase of the study. Dietary intake during the last 24 hours, including consumption of oil, was then estimated using standardized household measures (serving units). All dietary interviews were conducted in the home. To avoid possible reporting bias relating to supplement intake, the interviewers who conducted the 24-hour recall interviews were not involved in the distribution of oil or collection of other data. Dietary interviewers were frequently rotated between the intervention and the control villages.

Data on nutrient intake were calculated using a computerized food-composition table for Bangladeshi foods, which used nutrient data from regional food-composition databases (24,25). The computerized database was created and updated using the VBSedit and KOMEET software (Wageningen University, The Netherlands). The estimate of fat intake derived from all sources, including the supplemental fat, captured by 24-hour recall, was recorded as the total fat intake by the participants.

### Biochemical analysis

Blood samples each of 0.5 mL were collected in lithium heparinized microtainers either by finger-prick or ante-cubital venipuncture, immediately put on ice, shielded from light in a cold carrier, and transported to the field laboratory within 3-6 hours of collection. Plasma was then separated by centrifugation (3,000 rpm for 5 minutes) and transferred to labelled screw-top, amber-coloured cryovials and stored at -20 °C for 2-4 weeks in the field laboratory before being transferred to the central laboratory in Dhaka for storage at -7 °C until analysis 2-3 months later. Simultaneous determination of concentrations of serum retinol, lutein, and β-carotene was done by high-performance liquid chromatography (HPLC) in the Nutritional Biochemistry Laboratory of ICDDR,B (26). Retinol and carotenoids were quantified by determining the peak areas in HPLC chromatograms against standard curves. The lower limit of detection for retinol and carotenoids was 10 μg/L. Within-run coefficient of variation for retinol, lutein, and β-carotene was 1.0%, 2.0%, and 1.4% respectively. Between-run coefficient of variation for retinol, lutein, and β-carotene was 2-4%.

A nurse or a trained interviewer collected a casual breastmilk sample according to the method previously used in this population (27). Breastmilk (5 mL) was collected in a clean plastic container by manual expression by the mother between 9:00 and 15:30 hours from the breast that was not used for feeding the infant during the previous one hour or longer. All the samples were collected in the field and transported within 1-2 hours of collection to the field laboratory on ice and shielded from light in a cold box. Upon arrival at the field laboratory, the breastmilk samples were gently swirled 15-30 seconds to ensure thorough mixing and aliquots placed into amber-coloured, labelled cryovials for storage at -20 °C for 2-4 weeks before being transported to the central laboratory in Dhaka for storage at -70 °C until analysis 3-4 months later. Standard procedures were followed in collecting and handling breastmilk samples (28); the HPLC method was used for determining the concentration of breastmilk retinol (29).

### Analysis of data and statistics

Numeric variables were examined for their distribution outliers, and extreme values were identified and excluded from the analyses. Such values did not exceed 3-4% of the observations. Results are presented as mean±standard deviation for the normally-distributed variables or median and interquartile range for the variables not normally distributed. The differences between the groups were examined by Student's *t*-test for statistical significance. Changes in each of the outcome variables from baseline (pregnancy) to different follow-up periods were calculated for each subject by subtraction. Paired *t*-test was used for comparing within-group differences from baseline to the respective time periods. Independent sample *t*-tests were used for comparing changes at respective study periods between the groups. Concentrations of breastmilk retinol were compared between the intervention group and the control group by independent sample *t*-test of means. In this case, the change was not examined or tested as the intervention had been continuing for a certain period before the first sample of breastmilk was collected. The mean concentrations of infants’ plasma retinol, β-carotene, and lutein were compared between the groups by the independent sample *t*-test. The p value of <0.05 was considered statistically significant.

### Ethics

The Ethical Review Committee of ICDDR,B approved the study. An informed written consent was obtained from each woman before enrollment into the study.

## RESULTS

The baseline characteristics of 248 supplemented and 251 control women are presented in Table 1. The socioeconomic status, history of nightblindness, and nutritional status were comparable between the groups. Dietary intakes of carbohydrate, protein, and total energy were significantly higher in the control group than in the intervention group. During pregnancy, dietary fat contributed 6-8% of the total energy intake. The intervention group had a slightly higher fat intake compared to that of the control group, and the difference reached statistical significance. Both the groups had comparable but low mean concentrations of plasma retinol and high prevalence of low plasma retinol. Concentrations of both β-carotene and lutein were significantly higher in the control group than in the intervention group at baseline.

**Table 1.** Characteristics of study subjects at baseline (pregnancy)

Characteristics	Oil-supplemented group (mean±SD) (n=248)	Control group (mean±SD) (n=251)
Maternal age (years)	27±6*	26±5
Gestational age (weeks)	24±3	25±3
Parity	2.3±1.6	2.1±1.5
Maternal illiteracy (%)	40	41
History of nightblindness (%)	3.2	2.4
Income (x, 000 Taka)†,$	35 (20–49)	30 (20–49)
Anthopometric assessment		
Weight (kg)	45.2±5.5	45.1±5.4
Height (cm)	150.3±4.9	149.4±5.3
MUAC (mm)	227±18	227±18
BMI (kg/m^2^)	20.0±2.1	20.2±1.9
Dietary intake		
Carbohydrate (g/d)	298± 87	339±91*,**
Fat (g/d)	12±9	10±8*
Protein (g/d)	43±21	48±19**
Energy (kcal/d)	1378±367	1527±389**
β-carotene mg/d	852±1,029	990±1275
Plasma concentrations		
Retinol (mmol/L)	0.83±0.27	0.85±0.27
Retinol <0.70 mmol/L (%)	34	29
β-carotene mmol/L	0.16±0.07	0.17±0.07*
Lutein mmol/L	0.64±0.35	0.72±0.33**

†Median (25^th^-75^th^ percentile);

$1 US$=Tk 48 as in 1997;

*,**Significantly different from the other group:

**p<0.01;

*p<0.05;

BMI=Body mass index;

MUAC=Mid-upper arm circumference;

SD=Standard deviation

As expected, the mean fat intake from all the sources (including supplement) was significantly higher in the intervention group at 1, 3 and 6 months postpartum than that of the control group (Table 2). Fat intake in the control group increased slightly by six months postpartum measurement but it was not significantly different from earlier intakes. Supplementation with 18 g of oil resulted in doubling of fat intake in the intervention group compared to their baseline intake.

**Table 2.** Fat intake during the postpartum period

Sampling time	No.	Intervention mean±SD g/d	No.	Control mean±SD g/d
One month postpartum	125	21.1±12.5*	140	9.5±6.9
Three months postpartum	114	23.1±12.8*	97	9.3±6.2
Six months postpartum	164	24.6±11.2*	166	13.8±9.6

*Significantly higher than the control group (p<0.001);

SD=Standard deviation

**Fig. 2.**
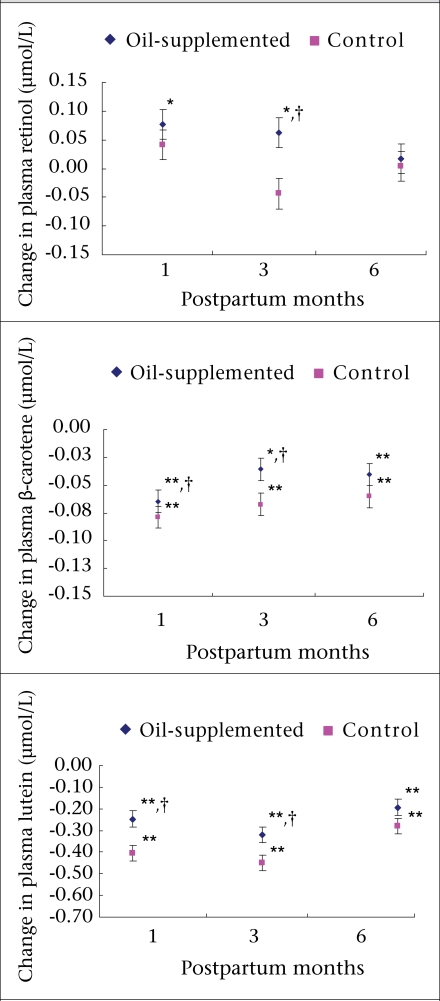
Changes in concentrations of post partum plasma retinol, β-carotene, and lutein compared to the pregnancy values between oil-supplemented and control women in rural Matlab, Bangladesh

Compared to the pregnancy (baseline) level, concentrations of plasma retinol increased in the intervention women in all the three measurement periods during the postpartum period (9%, 5%, and 0.4% at 1, 3 and 6 months postpartum); however, the change at one month postpartum reached statistical significance (Fig. 2). In the control group, compared to the pregnancy level, a small (∼5%) but a statistically non-significant increase in concentrations of plasma retinol was observed at one month postpartum whereas the mean changes at three months showed a statistically significant decrease (-8%) and a statistically non-significant decrease at six months postpartum. When the changes in concentrations of plasma retinol at different stages during postpartum were compared, the changes at three months postpartum in the intervention group was significantly different from that in the control group. The prevalence of vitamin A deficiency did not differ significantly between the groups, although it tended to be lower at one and Three months postpartum in the supplemented group.

**,* Significantly different from the pregnancy value (zero): **p<0.01, *p<0.05; †Significantly different from the control group: p<0.05

During the postpartum months, the concentration of plasma β-carotene declined compared to the pregnancy level in both intervention and control groups (Fig. 2). The decline in the intervention group was not statistically significant, except at six months postpartum when the highest decline in β-carotene concentration (-16%) was observed. In contrast, the control group experienced a substantial decline ranging from -23% to -28% of baseline value and was statistically significant at all the three measurements. When the changes (declines) between the groups were compared, the decline was significantly less in the intervention group at one month and three months postpartum while that at six months postpartum was also less in the intervention group but did not reach statistical significance.

The concentration of plasma lutein also declined during the postpartum measurements and followed the same trend as that observed in β-carotene (Fig. 2). However, compared to the pregnancy level, the within-group change (decline) in lutein concentrations during the postpartum period was significant in all the three measurements in both the study groups. When the changes in lutein concentration were compared between the intervention group and the control group, the decline was significantly less in the supplemented group, and the women comparatively maintained a significantly higher level of lutein at one month and three months postpartum but not at six months postpartum.

**Fig. 3.**
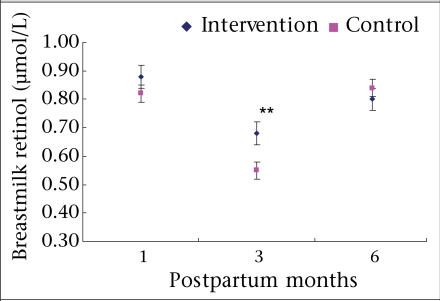
Mean concentrations of breastmilk retinol at 1, 3 and 6 months postpartum

The mean concentrations of breastmilk retinol were higher in the intervention group than those in the control group at one month and three months postpartum (0.06 and 0.13 μmol/L respectively), and the difference was significant at three months postpartum but not at six months postpartum (Fig. 3). No significant differences were observed in concentrations of plasma retinol, β-carotene, or lutein between infants born to the supplemented mothers and those born to the control mothers (Table 3).

**Table 3.** Concentrations of infants’ plasma retinol, β-carotene, and lutein at six months

Plasma component	Study group	No.	Mean±SD μmol/L
Retinol	Supplemented	54	0.50±0.15
	Control	66	0.48±0.17
β-carotene	Supplemented	15	0.10±0.01
	Control	19	0.10±0.01
Lutein	Supplemented	54	0.28±0.17
	Control	66	0.29±0.17

SD=Standard deviation

**Significantly different from the control group (p<0.01)

## DISCUSSION

The aim of this study was to examine the effect of supplementation of dietary fat on the vitamin A status of pregnant and lactating women and their infants in a population where vitamin A deficiency is prevalent and dietary fat intake is low. As indicated by the improvement in concentrations of blood retinol and carotenoids and the improvement in concentrations of breastmilk retinol in the supplemented group, these findings suggest that increasing the dietary fat intake may have beneficial effect on the vitamin A status of women during early lactation.

In this study, we aimed at increasing the fat intake by 18 g per day in the intervention group but the actual increase was less in many individual women. This was not unexpected since administration of the supplement was ultimately controlled by the study subjects themselves and not by the study team. Further, consumption of oil in this way, i.e. in a medicinal dosing, is not a normal dietary practice. Nonetheless, supplementation resulted in a doubling or greater fat intake in the intervention group.

The supplemented group increased concentrations of plasma retinol at 1, 3 and 6 months postpartum measurements (9%, 5%, and 4% above the baseline level) while an increase in the control group was observed only at one month postpartum measurement (5%) and a slightly declining tendency was noted at three and six months postpartum (-8 and -1%). The change compared to the baseline level at one month postpartum in the intervention was statistically significant, suggesting that dietary fat had a positive effect on plasma retinol concentration of the supplemented women. Both the groups showed lower concentrations of plasma β-carotene and lutein during postpartum; however, relatively less so in the supplemented group, indicating higher provitamin A bioavailability in the oil-supplemented women. These findings are consistent with earlier reports that fat supplementation in deficient population has beneficial effects on concentrations of retinol and carotenoids (18,30,31).

In the study population, dietary vitamin A is derived almost entirely from plant sources (6,32). Therefore, improvement in biochemical parameters in the dietary fat-supplemented group compared to the control group can be attributed to the improved bioavailability of provitamin A carotenoids and their subsequent conversion. Although only a small improvement in concentrations of vitamin A and carotenoids in the intervention was observed in this study, the absolute level of vitamin A was very low in both the groups and even lower than those reported from other developing countries (3). This may be due to overall lower dietary intake of provitamin A by Bangladeshi women (33) and possibly low intake of other essential nutrients relating to vitamin A metabolism, such as dietary protein and zinc in particular (34). Since the dietary source of vitamin A in this population is of plant origin, the absolute intake rarely meets the recommended daily allowance (RDA) during most of the year even when the higher conversion factors are assumed (33). Actual vitamin A content in diet in this population would be critically low if the recently-recommended and accepted conversion factors were applied (11,35). An earlier study in Indonesia reported a poorer plasma response in terms of improvement of retinol concentration when provitamin A caroteoids obtained from green-leafy vegetables were compared with purified β-carotene (12). Findings of the study raised concerns whether the strategy to promote the consumption of vegetables would have any significant role in ameliorating vitamin A deficiency. However, the study was not designed to evaluate the effect of increase in dietary fat on vitamin A and carotenoids status.

The women in the supplemented group of our study had relatively higher concentrations of breastmilk retinol at one month and three months postpartum than those in the control group. The increase in concentration of breastmilk retinol in the supplemented group translates into 8-18 μg of daily additional preformed retinol delivery in their infants (assuming a breastmilk intake of 700 mL per day), an amount equivalent to 4-10% of the basal requirements for infants up to the age of six months (8). The supplemented mothers had comparatively better vitamin A status during early lactation, a critically-important period for newborns (3,36,37). Any improvement in vitamin A content of breastmilk during early lactation, even if it is small, would be expected to have a significant impact in developing countries because exclusive breastfeeding is the greatest during this period. Results of a study in rural Bangladesh showed that breastmilk was the only source of vitamin A during infancy (33). This study also found that infants, aged 7-12 months, who were just weaned, had a daily intake of 3 retinol equivalents per day of vitamin A and concluded that infants in Bangladesh have virtually no other source of vitamin A than breastmilk during the first year of their life.

Our findings are consistent with those of other studies in developing countries where the breastmilk retinol level is either deficient or at the marginal range (3,38). Of interest, the breastmilk retinol found in our rural population was lower than that of a disadvantaged urban population in Bangladesh (39). This difference might be due to possible greater access of the urban population to foods of animal origin and higher intake of dietary fat than the rural population in Bangladesh.

We have observed a substantial fluctuation in concentrations of breastmilk retinol. Such fluctuations in concentrations of breastmilk retinol have also been reported by others (40). It is also known that concentration of breastmilk retinol is highly dependent on concentration of fat in breastmilk (41). Further, fat is the most variable macronutrient in human milk and reflects maternal diet (42).

No difference was observed in concentrations of the infants’ plasma retinol, β-carotene, or lutein. Possible reason might be that vitamin A status of the infants was measured at the end of six months of age when the difference in maternal vitamin A status between the supplemented and the control mothers did not exist. It might also be possible that the benefit to the infants of the intervention mothers, if it had occurred in early infancy, might not have been large enough to maintain a sustained higher concentration up to six months of age. Although the intervention mothers had a better concentration of breastmilk retinol during early lactation, it is notable that retinol concentration in most women of both the groups was always less than 1.05 μmol/L, the concentration considered to be just enough to meet the basal requirement of the infant but not enough to build good vitamin A stores (5).

### Conclusions

We conclude that a small amount of dietary fat supplementation during pregnancy and lactation has a beneficial effect on maternal vitamin A status during lactation in populations where plant sources are the major contributors to dietary vitamin A intake and the fat intake is extremely low. However, it is notable that the mean concentrations of plasma retinol in this population were far below than those in developed countries (43,44) and even lower than those reported from other developing countries (38,45,46). Therefore, it is highly probable that this population requires multiple interventions, including long-term target of increased consumption of vitamin A-containing foods, in addition to the promotion of the increased intake of dietary fat and postpartum supplementation of high-dose vitamin A to ensure adequate vitamin A status of mothers and infants.

## ACKNOWLEDGEMENTS

The study was conducted at ICDDR,B with support from a grant USAID/OMNI HNI's CA No. HRN-5122-A-00-3046-00-(“CA”). ICDDR,B acknowledges with gratitude the commitment of USAID/OMNI to the Centre's research efforts. Financial support from the Division of Human Nutrition and Epidemiology, Wageningen, is gratefully acknowledged. The authors thank all the mother-infant pairs for their valuable participation in the study.
